# Stress Tolerance and Contribution to Aroma Profile of *Pichia kudriavzevii* GAAS-JG-1 Isolated from Apricot Fermentation in Co-Fermentation of Sea Buckthorn Wine

**DOI:** 10.3390/microorganisms13071491

**Published:** 2025-06-26

**Authors:** Yuwen Mu, Yu’an Wang, Chaozhen Zeng

**Affiliations:** Institute of Agricultural Products Storage and Processing, Gansu Academy of Agricultural Sciences, Lanzhou 730070, China; muyw@gsagr.cn (Y.M.); gswya@gsagr.cn (Y.W.)

**Keywords:** *Pichia kudriavzevii*, environmental tolerance, co-fermentation, aroma compounds, sea buckthorn wine

## Abstract

High-acidity fruit wines, such as sea buckthorn wine, are valued for their nutritional benefits but often suffer from excessive tartness and limited aroma complexity, which restrict their consumer acceptance. The application of non-Saccharomyces yeasts with acid tolerance and flavor-enhancing potential offers a promising strategy to address these challenges. In this study, a highly acid-tolerant yeast strain, *Pichia kudriavzevii* GAAS-JG-1, was isolated from a naturally fermented apricot system and systematically characterized in terms of its taxonomy, physiological properties, and fermentation potential. The experimental results demonstrated that *Pichia kudriavzevii* GAAS-JG-1 maintained robust growth activity (OD_600_ = 1.18 ± 0.09) even under extremely acidic conditions (pH 2.0). Furthermore, the strain exhibited a strong tolerance to high ethanol concentrations (16%), elevated sugar levels (350 g/L), and substantial sulfur dioxide exposure (500 mg/L). Optimal growth was observed at 35 °C (OD_600_ = 2.21 ± 0.02). When co-fermented with *Saccharomyces cerevisiae* in sea buckthorn wine, the ethyl acetate content increased significantly from 303.71 μg/L to 4453.12 μg/L, while the ethyl propionate levels rose from 5.18 μg/L to 87.75 μg/L. Notably, *Pichia kudriavzevii* GAAS-JG-1 also produced novel flavor compounds such as methyl acetate and ethyl 3-methylthiopropionate, which were absent in the single-strain fermentation. These findings highlight the potential of *Pichia kudriavzevii* GAAS-JG-1 as a valuable non-*Saccharomyces* yeast resource with promising applications in the fermentation of high-acidity specialty fruit wines.

## 1. Introduction

Fruit wine brewing is a complex microbial metabolic process in which the intermediate and final products generated during fermentation play a crucial role in determining the aroma, flavor, and overall quality of the wine [1]. In addition to the chemical composition of the raw materials, the yeast species and their metabolic characteristics also profoundly influence fruit wine quality [2]. During fermentation, various compounds from the raw materials are released and transferred into the wine, with many key aroma compounds being formed under the metabolic activity of yeast [3]. Currently, *Saccharomyces cerevisiae* is widely employed in fruit wine production due to its excellent fermentation performance and strong ethanol tolerance [4]. However, in the production of high-acidity fruit wines (pH 2.5–3.0), the growth of *S. cerevisiae* is inhibited below pH 3.0, resulting in reduced fermentation activity and a decline in wine flavor complexity and sensory quality [5]. Consequently, under high-acidity conditions, the fermentation performance of *S. cerevisiae* may be restricted, ultimately affecting the final quality of the fruit wine. Therefore, screening and selecting yeast strains with superior acid tolerance has become a critical research focus for improving the flavor and quality of high-acidity fruit wines.

In recent years, the selection and application of acid-tolerant non-*Saccharomyces* yeasts have emerged as an effective strategy for optimizing the production of high-acidity fruit wines. Non-*Saccharomyces* yeasts have garnered increasing attention due to their ability to enhance flavor complexity and modulate aroma profiles [6,7,8]. Different non-*Saccharomyces* yeasts typically exhibit distinct metabolic advantages. For instance, *Torulaspora delbrueckii* and *Metschnikowia pulcherrima* are known for their capacity to regulate ester and higher alcohol metabolism, thereby enhancing fruity and floral characteristics [9,10]. Among these, *Pichia kudriavzevii* has been identified as a highly adaptable non-*Saccharomyces* yeast that can maintain robust growth activity under acidic conditions (pH 3.2–4.0) [11]. Studies have demonstrated that *Pichia kudriavzevii* optimizes the flavor profile of fruit wines through specific metabolic pathways [12], promoting the accumulation of esters such as ethyl acetate and ethyl hexanoate, while reducing higher alcohols (e.g., isoamyl alcohol, phenylethanol) and volatile acids (e.g., acetic acid, hexanoic acid), thus contributing to a more balanced and harmonious wine profile [13,14].

In fruit wines, ester compounds are crucial determinants of their aroma and flavor profiles [15,16]. Non-*Saccharomyces* yeasts can influence ester synthesis and degradation by regulating the expression of esterases or acetyltransferases [17,18]. In white wine and cider systems, non-*Saccharomyces* yeasts such as *Hanseniaspora uvarum* and *Starmerella bacillaris* have been shown to enhance the production of key esters, including ethyl acetate, ethyl octanoate, and phenethyl acetate, thereby intensifying fruity and floral characteristics [19,20]. Moreover, fermentation strategies such as co-fermentation and sequential fermentation can shape distinct metabolic pathways and flavor profiles. For example, using non-*Saccharomyces* yeasts during the initial fermentation stage to promote ester synthesis, followed by *S. cerevisiae* to complete sugar consumption, can effectively balance fermentation efficiency and the accumulation of aroma compounds [21,22,23].

Although *Pichia kudriavzevii* has gradually gained applications in the food and beverage industries, research on its use in high-acidity fruit wines remains limited, particularly regarding its influence on aroma metabolism under extreme fermentation conditions. Sea buckthorn wine, known for its nutritional value and unique sensory characteristics, often suffers from excessive tartness and astringency due to its high acidity, which negatively impacts consumer acceptance and market potential. Therefore, there is an urgent need to select and develop yeast strains capable of tolerating harsh fermentation conditions while improving the flavor profile of high-acidity fruit wines.

In this study, the aim was to screen and characterize an acid-tolerant yeast strain with potential application in the fermentation of high-acidity fruit wines, and to evaluate its fermentation characteristics and impact on the flavor profile of sea buckthorn wine. In this context, an acid-tolerant *Pichia kudriavzevii* GAAS-JG-1 was selected for further investigation. It should be noted that this study focused on the screening and characterization of this particular strain, while other commercial non-*Saccharomyces* yeasts were used solely as reference strains for comparative purposes, rather than for comprehensive biodiversity analysis.

## 2. Materials and Methods

### 2.1. Preparation and Spontaneous Fermentation of Apricot Puree

Fully ripened and intact apricots were carefully selected, thoroughly cleaned, and subsequently pitted. The fruit flesh was homogenized into a uniform puree using a precision homogenizer. To preserve the integrity of the fruit’s native microbial community, the puree was deliberately not subjected to sterilization procedures. After preparation, the fresh puree was subjected to spontaneous fermentation under suitable conditions. Upon completion of the spontaneous fermentation, samples from the fermented system were collected for the microbial isolation procedure.

### 2.2. Isolation and Purification of Strains

A 10 g sample of spontaneously fermented apricot puree (Tianshui, China) was aseptically weighed and transferred into 90 mL sterile physiological saline (0.85% NaCl) (Guangzhou, China). The mixture was homogenized thoroughly using a homogenizer (Shanghai, China) and subjected to serial dilution (10^−1^ to 10^−6^). Subsequently, aliquots (0.1 mL) of each dilution were spread evenly onto YPD agar plates (purchased from Qingdao Haibo Biotechnology Co., Ltd., Qingdao, China) with pH adjusted to 3.0 using citric acid (Tianjin, China)or lactic acid (Tianjin, China)to selectively isolate acid-tolerant yeast strains. Plates were incubated at 28 °C for 48–72 h. Colonies exhibiting distinctive morphological characteristics, pigmentation, and textures were selected and transferred onto WL nutrient agar medium (purchased from Qingdao Haibo Biotechnology Co., Ltd., Qingdao, China) for differential purification. The streaking purification procedure was repeated 2–3 times to obtain pure, homogeneous colonies. The total number of colonies isolated from the apricot puree was approximately 10^6^ CFU/g. Purified yeast strains were inoculated onto YPD slants for short-term storage or preserved long-term at −80 °C as suspensions containing 20% glycerol (Tianjin, China).

### 2.3. Identification of Pichia kudriavzevii GAAS-JG-1

Morphological Identification: The purified strain was streak-inoculated onto WL nutrient agar medium and incubated inversely at 28 °C for 5 days. Upon completion of the incubation, the colony morphology of *Pichia kudriavzevii* GAAS-JG-1 was observed and recorded, including colony size, color, and surface characteristics. Additionally, digital photographs were taken to document the colony morphology.

Molecular Identification: Genomic DNA of *Pichia kudriavzevii* GAAS-JG-1 was extracted using a commercial genomic DNA extraction kit. The extracted DNA served as a template for the amplification of the D1/D2 variable region of the 26S rDNA using the universal primers NL1 (5′-GCATATCAATAAGCGGAGGAAAAG-3′) and NL4 (5′-GGTCCGTGTTTCAAGACGG-3′). The PCR products were sent to Sheng gong Biotech Co., Ltd. (Shanghai, China) for bidirectional sequencing. The obtained sequences were analyzed for homology using the NCBI BLAST (version 2.13.0) online tool (BLASTN) against the NCBI NR/NT database. The taxonomic status of the strain was determined based on sequence identity (≥99%) and query coverage (≥95%).

### 2.4. Preservation of Pichia kudriavzevii GAAS-JG-1

The *Pichia kudriavzevii* GAAS-JG-1 strain was officially deposited at the China General Microbiological Culture Collection Center (CGMCC) on 17 March 2023, with the accession number CGMCC No. 26816. The CGMCC is affiliated with the Institute of Microbiology, Chinese Academy of Sciences (IMCAS), located at No. 3, Yuan 1, Beichen West Road, Chaoyang District, Beijing, China, with the postal code 100101.

### 2.5. Tolerance Assays of Yeast Strains

Tolerance assays were conducted using activated seed cultures of *Pichia kudriavzevii* GAAS-JG-1 and three control non-Saccharomyces yeast strains, namely, *Torulaspora delbrueckii* CICC 1004, *Pichia anomala* CICC 31838, and *Wickerhamomyces anomalus* CICC 31399. The control strains were commercially available reference strains used only for performance comparison in this study; no further identification or characterization was performed on them, since the primary aim of this study was to highlight the fermentation potential of the isolated strain *Pichia kudriavzevii* GAAS-JG-1. All cultures were inoculated at a volume of 1 mL into 10 mL sterile YPD medium and incubated statically at 28 °C for 72 h, unless otherwise indicated. Post-incubation, optical density at 600 nm (OD_600_) was measured. Samples with high OD values were diluted appropriately before measurement, and actual OD values were calculated by multiplying the measured values by the dilution factor.

#### 2.5.1. pH Tolerance

The pH of YPD medium was adjusted to 2.0, 3.0, 4.0, 5.0, and 6.0 using sterile citric acid solution before inoculation and incubation as described above.

#### 2.5.2. Ethanol Tolerance

Anhydrous ethanol was aseptically added to sterile YPD medium to achieve final concentrations of 8%, 10%, 12%, 14%, and 16% (v/v) prior to inoculation and incubation.

#### 2.5.3. Glucose Tolerance

Glucose was aseptically added to sterile YPD medium to reach final concentrations of 150, 200, 250, 300, and 350 g/L before inoculation and incubation.

#### 2.5.4. Sulfur Dioxide Tolerance

Potassium metabisulfite (K_2_S_2_O_5_) was aseptically added to sterile YPD medium to obtain sulfur dioxide (SO_2_) concentrations of 100, 200, 300, 400, and 500 mg/L prior to inoculation and incubation.

#### 2.5.5. Temperature Tolerance

Inoculated YPD medium cultures were incubated statically at different temperatures of 15 °C, 25 °C, and 35 °C for 72 h, after which OD_600_ measurements were performed as previously described.

#### 2.5.6. Laboratory-Scale Fermentation of Sea Buckthorn Fruit Wine

The experiment employed *Pichia kudriavzevii* GAAS-JG-1 for sea buckthorn fruit wine fermentation, with *Saccharomyces cerevisiae* (CICC 32168) monoculture fermentation serving as the control. Mature sea buckthorn berries were washed, destemmed, crushed, and pressed to obtain juice, which was filtered to obtain clarified juice. The juice was pasteurized at 121 °C for 15 min prior to fermentation. The sugar content was adjusted to 150 g/L and the pH was adjusted to 2.83. Potassium metabisulfite (100 mg/L) and pectinase (0.6%) were added to improve juice clarity and fermentation stability. Fermentations were conducted in 500 mL sterilized glass fermentation bottles containing 300 mL of sea buckthorn juice. The experimental group was subjected to a sequential inoculation strategy: *Pichia kudriavzevii* GAAS-JG-1 was first inoculated at a concentration of 1 × 10^6^ cfu/mL with an inoculation volume of 6% (v/v). After 24 h, *S. cerevisiae* (CICC 32168) was subsequently inoculated under the same conditions. The fermentation was conducted statically at 20 °C. In the control group, *S. cerevisiae* (CICC 32168) was used as a monoculture starter and fermented under identical conditions. Fermentation progress was monitored by daily weighing of the fermentation vessels. The fermentation was considered complete when no further weight loss due to CO_2_ release was observed for two consecutive days. The experiment focused on evaluating the effect of *Pichia kudriavzevii* GAAS-JG-1 on the volatile aroma profile of sea buckthorn fruit wine during sequential fermentation.

#### 2.5.7. Analysis of Volatile Aroma Compounds in Sea Buckthorn Fruit Wine

The determination of volatile aroma compounds in different fruit wines was performed using headspace solid-phase microextraction coupled with gas chromatography-mass spectrometry (HS-SPME-GC-MS), based on the method described by Zeng et al. (2025) [24], with modifications for optimization.

Sample Preparation: The sample pretreatment was conducted using the headspace solid-phase microextraction method. A 5 mL wine sample was mixed with 1 g of sodium chloride and 50 μL of internal standard (3-octanol) in a 15 mL headspace vial. After vortex mixing, the vial was kept in a refrigerator at 4 °C overnight. The sample vials were processed using a TriPlus RSH Autosampler-SPME system with a 50/30 μm DVB/CAR/PDMS fiber. The extraction conditions were set as follows: adsorption at 60.0 °C for 30 min, followed by a desorption period of 5 min.

GC Analysis Conditions: GC-MS analyses were carried out on a TRACE 1310 gas chromatograph coupled with an ISQ-LT mass spectrometer (Thermo Fisher Scientific, Waltham, MA, USA). A DB-WAX column (30 m × 0.25 mm × 0.25 μm) was used for separation. The injector temperature was 250 °C, and helium was used as the carrier gas at a constant flow rate of 1.2 mL/min. The injection volume was 1 μL in split mode with a split ratio of 40:1. The oven temperature program was as follows: initial temperature 40 °C for 3 min; increased at 6 °C/min to 180 °C (held for 2 min); then increased at 10 °C/min to 230 °C (held for 6 min).

MS Detection Conditions: Mass spectrometry (MS) was conducted using an electron impact (EI) ionization source at an electron energy of 70 eV. The ion source temperature was set at 200 °C, and the interface temperature was maintained at 250 °C. The mass scan range was set between 33.00 and 450.00 amu.

Identification and Quantification of Volatile Compounds: Volatile compounds were presumptively identified by comparing their mass spectra with entries in the NIST Mass Spectral Library (NIST 17) using Thermo Xcalibur (version 4.1) software. A similarity threshold of 85% was applied for tentative identification. As no authentic reference standards were injected and retention indices were not calculated, all identifications are considered presumptive. Quantification was performed using the internal standard method, with 3-octanol as the internal standard. The concentration of each compound was calculated based on the ratio of its peak area to that of the internal standard, assuming similar response factors for compounds of similar chemical nature. Results were expressed as micrograms per liter (μg/L) of wine. Each sample was analyzed in triplicate.

### 2.6. Statistical Analysis

The results were expressed as the mean ± standard deviation. Univariate analysis of variance (ANOVA) of the data and the significance of the difference test was performed using SPSS 24.0 software. The LSD letter labeling was used to indicate significance. Graphical representations were created using Origin 2021 software.

## 3. Results and Discussion

### 3.1. Isolation and Identification of Pichia kudriavzevii GAAS-JG-1

**Figure 1:**
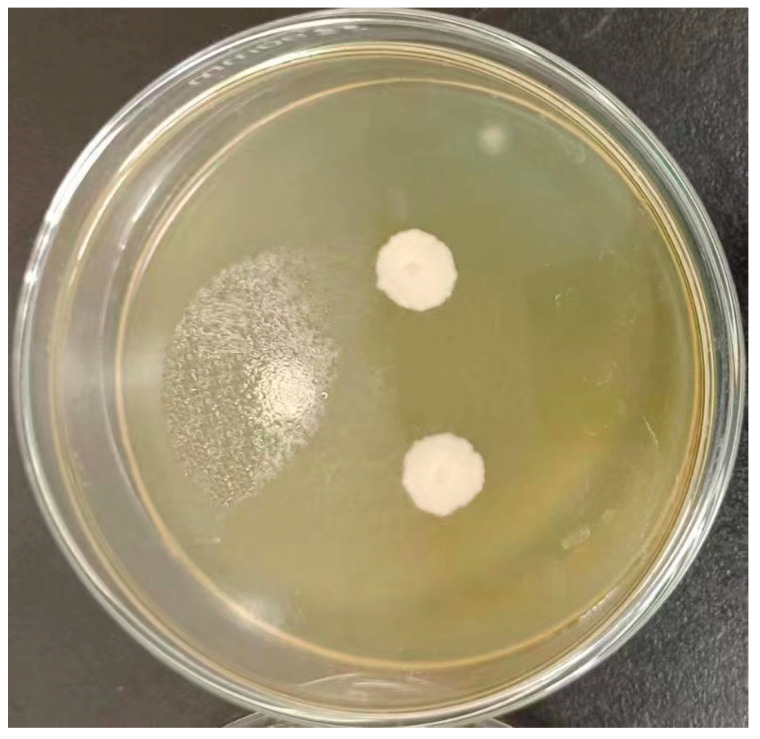
Colony morphological characteristics of *Pichia kudriavzevii* GAAS-JG-1.

During the natural fermentation process of apricots, a yeast strain was isolated and designated as strain *Pichia kudriavzevii* GAAS-JG-1 based on its morphological characteristics (Figure 1). The 26S rDNA sequence of strain *Pichia kudriavzevii* GAAS-JG-1 was amplified and sequenced, with the sequencing result presented in Table 1. The obtained sequence was analyzed using the NCBI BLAST online tool, which showed 100.0% identity and 100% query coverage with *Pichia kudriavzevii* isolate K44 (GenBank accession no. MW916140.1), representing the top hit among the BLAST results. No other closely related species showed higher similarity. Based on these identification results, the strain was confirmed to belong to *Pichia kudriavzevii* and was designated as *Pichia kudriavzevii* GAAS-JG-1. The identified strain *Pichia kudriavzevii* GAAS-JG-1 was deposited at the China General Microbiological Culture Collection Center (CGMCC) on March 17, 2023, under the accession number CGMCC No. 26816. The storage location is the Institute of Microbiology, Chinese Academy of Sciences, No. 3, Yuan 1, Beichen West Road, Chaoyang District, Beijing, China; postal code: 100101.

### 3.2. Physiological Characterization

This section systematically evaluated the physiological tolerance characteristics of *Pichia kudriavzevii* GAAS-JG-1 under various stress conditions, including low pH, high ethanol, high sugar, sulfur dioxide, and temperature. For comparative analysis, three commercial non-*Saccharomyces* reference strains (CICC 1004, CICC 31838, and CICC 31399) were used as control strains throughout all tolerance tests. The comparative results are presented below.

#### 3.2.1. Properties of pH Tolerance of the Selected Yeasts

**Figure 2:**
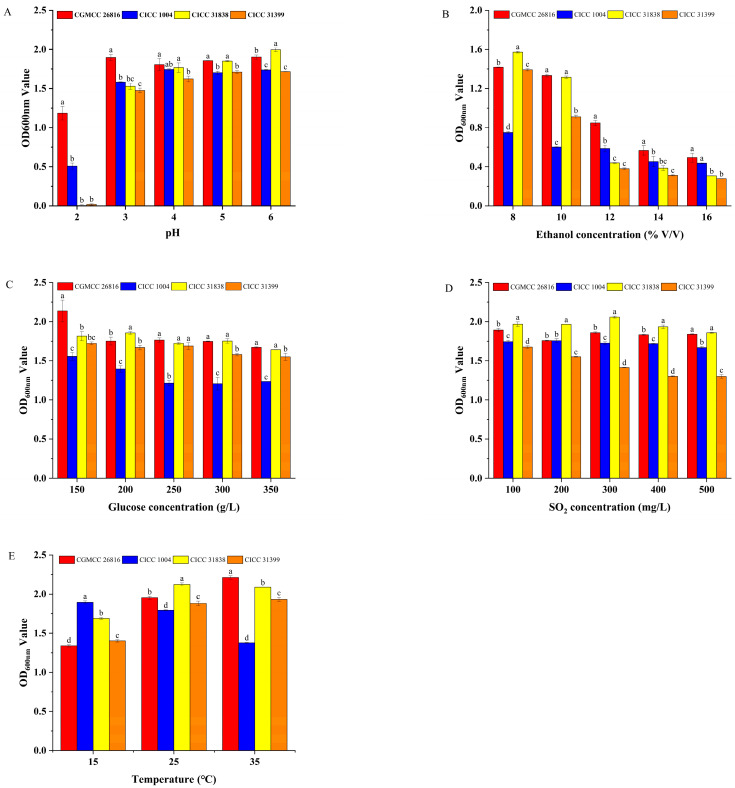
(**A**) Comparison of pH tolerance between Pichia kudriavzevii GAAS-JG-1 (CGMCC No. 26816) and Other Non-*Saccharomyces* Yeasts. (**B**) Comparison of Ethanol tolerance between *Pichia kudriavzevii* GAAS-JG-1 (CGMCC No. 26816) and Other Non-*Saccharomyces* Yeasts. (**C**) Comparison of glucose tolerance between *Pichia kudriavzevii* GAAS-JG-1 (CGMCC No. 26816) and Other Non-*Saccharomyces* Yeasts. (**D**) Comparison of sulfur dioxide tolerance between *Pichia kudriavzevii* GAAS-JG-1 (CGMCC No. 26816) and Other Non-*Saccharomyces* Yeasts. (**E**) Comparison of temperature tolerance between *Pichia kudriavzevii* GAAS-JG-1 (CGMCC No. 26816) and Other Non-*Saccharomyces* Yeasts.

The low pH tolerance of the tested strains was assessed under varying pH conditions. The results are shown in Figure 2A. The experimental results demonstrated that *Pichia kudriavzevii* GAAS-JG-1 exhibited significantly superior growth ability compared to the control strains under all tested pH conditions. When the pH dropped to 2, the OD_600_ value of *Pichia kudriavzevii* GAAS-JG-1 reached 1.18 ± 0.09, whereas the OD_600_ values of the control strains were 0.51 ± 0.04, 0.57 ± 0.01, and 0.49 ± 0.01, respectively, indicating that *Pichia kudriavzevii* GAAS-JG-1 possesses strong tolerance to extreme acidity. Moreover, as the pH increased, the growth ability of *Pichia kudriavzevii* GAAS-JG-1 gradually improved, maintaining relatively high and stable OD_600_ values (1.81–1.90) within the pH range of 3–6. In contrast, the control strains exhibited significantly inhibited growth under pH 2–3 conditions, with OD_600_ values below 1.6. These results suggest that *Pichia kudriavzevii* GAAS-JG-1 possesses excellent adaptability to low-pH conditions. Even in an extremely acidic environment (pH 2), *Pichia kudriavzevii* GAAS-JG-1 maintained a robust growth performance, which was notably superior to the control strains. This finding highlights the suitability of *Pichia kudriavzevii* GAAS-JG-1 for improving the fermentation of high-acidity fruit wines. The ability of *Pichia kudriavzevii* GAAS-JG-1 to tolerate extremely low-pH conditions (as low as pH 2) provides theoretical support for its industrial application in acidic environments. Additionally, this characteristic suggests that *Pichia kudriavzevii* GAAS-JG-1 may possess unique physiological regulatory mechanisms to cope with acid stress, as also reported in previous studies highlighting the acid tolerance of *Pichia kudriavzevii* strains in fruit wine fermentations [11,12].

#### 3.2.2. Properties of Ethanol Tolerance of the Selected Yeasts

The ethanol tolerance of the tested strains was evaluated under ethanol concentrations ranging from 8% to 16%. The results are shown in Figure 2B. Within the tested ethanol concentration range (8–16%), the growth of all strains was inhibited to varying degrees as the ethanol concentration increased. However, *Pichia kudriavzevii* GAAS-JG-1 exhibited superior ethanol tolerance across the entire concentration range, particularly at higher ethanol concentrations, where its OD_600_ values were significantly higher than those of the control strains. Specifically, at 8% ethanol concentration, the OD_600_ value of *Pichia kudriavzevii* GAAS-JG-1 was 1.42 ± 0.01, slightly lower than that of CICC 31838 (1.57 ± 0.01) but significantly higher than those of the other control strains (*p* < 0.05). As the ethanol concentration increased to 12–16%, the growth advantage of *Pichia kudriavzevii* GAAS-JG-1 became more pronounced. At 12% ethanol, *Pichia kudriavzevii* GAAS-JG-1 achieved an OD_600_ value of 0.85 ± 0.02, while the OD_600_ values of the control strains CICC 1004, CICC 31838, and CICC 31399 were 0.59 ± 0.02, 0.44 ± 0.01, and 0.38 ± 0.01, respectively, with significant differences observed (*p* < 0.05). Notably, at 16% ethanol concentration, *Pichia kudriavzevii* GAAS-JG-1 maintained measurable growth with an OD_600_ value of 0.49 ± 0.03, whereas the OD_600_ values of the control strains were all below 0.45, further demonstrating *Pichia kudriavzevii* GAAS-JG-1′s remarkable ethanol tolerance advantage in high-alcohol environments. Such ethanol tolerance demonstrates the promising potential of *Pichia kudriavzevii* GAAS-JG-1 for applications in high-alcohol fermentation systems. Moreover, this tolerance characteristic may be related to *Pichia kudriavzevii* GAAS-JG-1′s unique metabolic mechanisms and cell membrane structure, as also observed in previous studies of ethanol-tolerant *Pichia kudriavzevii* strains [11,13].

#### 3.2.3. Properties of Glucose Tolerance of the Selected Yeasts

The glucose tolerance of the tested strains was evaluated under glucose concentrations of 150 g/L, 200 g/L, 250 g/L, 300 g/L, and 350 g/L. The results are shown in Figure 2C. As the glucose concentration increased, the growth of all strains was inhibited to some extent. However, *Pichia kudriavzevii* GAAS-JG-1 exhibited a significantly better growth performance in the high-glucose environments compared to the reference strains. At a glucose concentration of 150 g/L, the OD_600_ value of *Pichia kudriavzevii* GAAS-JG-1 was 2.14 ± 0.14, significantly higher than those of all reference strains (OD_600_ values ranging from 1.56 ± 0.05 to 1.81 ± 0.06, *p* < 0.05). When the glucose concentration increased to 200 g/L and 250 g/L, *Pichia kudriavzevii* GAAS-JG-1 maintained a strong growth performance, with OD_600_ values of 1.75 ± 0.04 and 1.77 ± 0.02, respectively, demonstrating significant glucose tolerance (*p* < 0.05). In contrast, the reference strain CICC 1004 showed weaker growth under the same conditions, with OD_600_ values of 1.40 ± 0.03 and 1.21 ± 0.02, respectively. At extremely high glucose concentrations (350 g/L), *Pichia kudriavzevii* GAAS-JG-1 continued to exhibit relatively stable growth, achieving an OD_600_ value of 1.67 ± 0.01, which was comparable to that of CICC 31838 (1.64 ± 0.01) and significantly higher than those of CICC 1004 (1.23 ± 0.01) and CICC 31399 (1.55 ± 0.03). These results suggest that *Pichia kudriavzevii* GAAS-JG-1 could serve as a valuable strain for enhancing fermentation efficiency under high-sugar conditions. Furthermore, *Pichia kudriavzevii* GAAS-JG-1′s glucose tolerance may be associated with its unique metabolic pathways, osmoregulatory mechanisms, and cell membrane properties, as also reported in previous studies on *P. kudriavzevii* [12,13].

#### 3.2.4. Properties of Sulfur Dioxide Tolerance of the Selected Yeasts

The sulfur dioxide tolerance of the tested strains was evaluated under SO_2_ concentrations ranging from 100 mg/L to 500 mg/L. The results are shown in Figure 2D. Within this concentration range, both *Pichia kudriavzevii* GAAS-JG-1 and the reference strains demonstrated strong sulfur dioxide tolerance, yet significant differences were observed among the strains. At a lower SO_2_ concentration (100 mg/L), CICC 31838 exhibited the highest OD_600_ value (1.97 ± 0.02), while the OD_600_ value of *Pichia kudriavzevii* GAAS-JG-1 was 1.89 ± 0.02, slightly lower than CICC 31838 but significantly higher than CICC 1004 (1.74 ± 0.01) and CICC 31399 (1.68 ± 0.01) (*p* < 0.05). As the SO_2_ concentration increased to 200 mg/L and 300 mg/L, *Pichia kudriavzevii* GAAS-JG-1 maintained a strong growth performance, with OD_600_ values of 1.76 ± 0.01 and 1.86 ± 0.01, respectively. These values were significantly higher than those of CICC 1004 (1.76 ± 0.02 and 1.73 ± 0.02) and CICC 31399 (1.55 ± 0.01 and 1.41 ± 0.01) (*p* < 0.05). At a high SO_2_ concentration of 500 mg/L, *Pichia kudriavzevii* GAAS-JG-1 achieved an OD_600_ value of 1.84 ± 0.01, comparable to that of CICC 31838 (1.86 ± 0.01) (*p* > 0.05), yet significantly higher than those of CICC 1004 (1.67 ± 0.01) and CICC 31399 (1.30 ± 0.02) (*p* < 0.05). Its strong sulfur dioxide tolerance underlines the competitive advantage of *Pichia kudriavzevii* GAAS-JG-1 in SO_2_-rich fermentation environments. Moreover, this strong sulfur dioxide tolerance reflects the strain’s physiological advantage in coping with fermentation stress, as also reported for *P. kudriavzevii* and other non-*Saccharomyces* yeasts used in winemaking [14,15].

#### 3.2.5. Properties of Temperature Tolerance of the Selected Yeasts

The temperature tolerance of the tested strains was evaluated under conditions of 15 °C, 25 °C, and 35 °C. The results are shown in Figure 2E. Significant differences in growth performance were observed among the strains at different temperatures (*p* < 0.05). At a low temperature (15 °C), CICC 1004 exhibited the highest OD_600_ value (1.90 ± 0.02), which was significantly higher than that of the other strains. The OD_600_ value of *Pichia kudriavzevii* GAAS-JG-1 was 1.34 ± 0.01, slightly lower than those of CICC 31838 and CICC 31399 (*p* < 0.05). As the temperature increased to 25 °C, the growth performance of *Pichia kudriavzevii* GAAS-JG-1 improved significantly, with an OD_600_ value of 1.95 ± 0.01, second only to CICC 31838 (2.12 ± 0.02), and higher than CICC 1004 (OD_600_ = 1.58 ± 0.03) and CICC 31399 (OD_600_ = 1.86 ± 0.01) (*p* < 0.05). At a higher temperature (35 °C), *Pichia kudriavzevii* GAAS-JG-1 achieved its highest growth performance, with an OD_600_ value of 2.21 ± 0.02, significantly higher than all the control strains (*p* < 0.05). CICC 31838 followed with an OD_600_ value of 2.09 ± 0.01, while CICC 1004 and CICC 31399 exhibited weaker growth, with OD_600_ values of 1.38 ± 0.01 and 1.93 ± 0.02, respectively. In conclusion, *Pichia kudriavzevii* GAAS-JG-1 demonstrated strong adaptability to varying temperature conditions, maintaining good growth performance at moderate temperatures (25 °C) and achieving optimal biomass at elevated temperatures (35 °C). The remarkable temperature adaptability of *Pichia kudriavzevii* GAAS-JG-1 confirms its suitability for fermentation processes conducted across a wide temperature range. Notably, its superior performance at higher temperatures underscores its physiological adaptability, as also reported in previous studies on *P. kudriavzevii* in diverse fermentation environments [11,13].

### 3.3. The Effect of Pichia kudriavzevii GAAS-JG-1 on Volatile Flavor Compounds in Fermented Sea Buckthorn Wine

**Figure 3:**
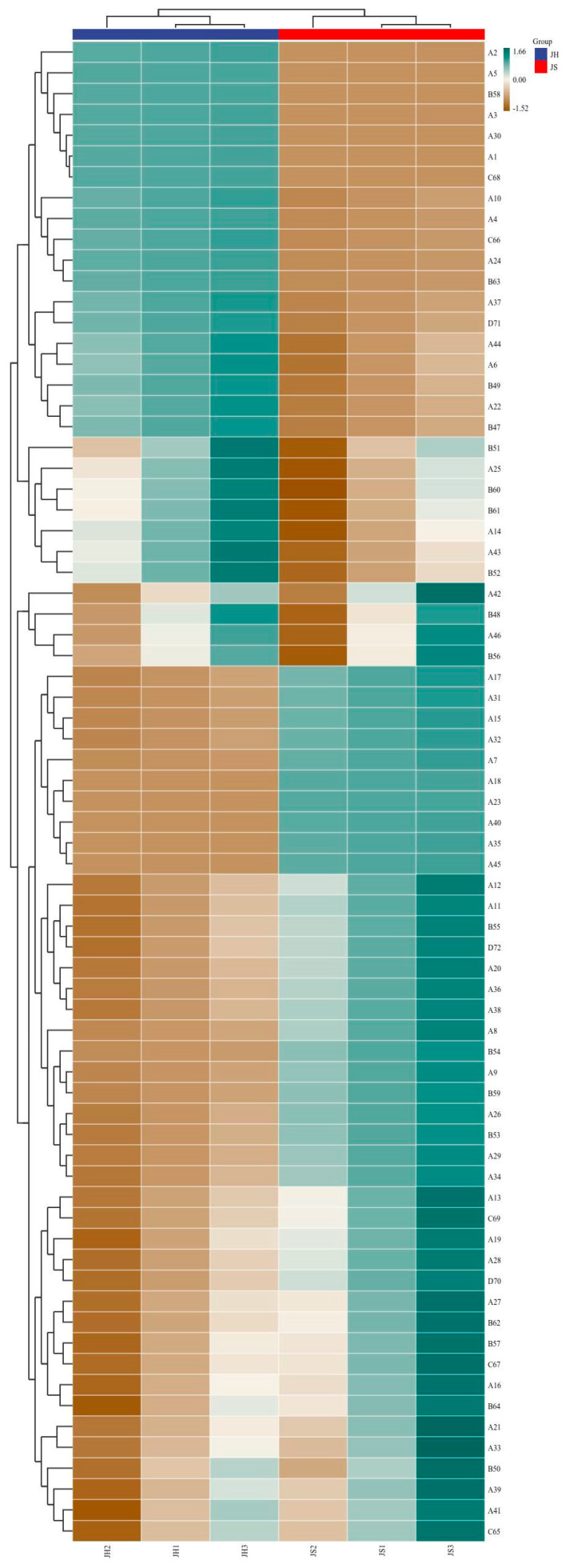
Heatmap cluster analysis of volatile flavor compounds in sea buckthorn wine under different fermentation conditions. JS: Monoculture fermentation of *Saccharomyces cerevisiae* (CICC 32168); JH: *Saccharomyces cerevisiae* (CICC 32168) was inoculated initially, and *Pichia kudriavzevii* GAAS-JG-1 was inoculated after 24 h.

Volatile flavor compounds are key components that contribute to the characteristic flavor profile of fermented sea buckthorn wine, exerting a decisive influence on its sensory quality. In this study, the volatile compounds in sea buckthorn wines produced via two fermentation modes were systematically analyzed: JS (single fermentation with *Saccharomyces cerevisiae* CICC 32168) and JH (co-fermentation with *S. cerevisiae* CICC 32168 and *Pichia kudriavzevii* GAAS-JG-1). A total of 48 esters, 18 alcohols, 7 acids, 3 aldehydes/ketones, and a small number of other flavor compounds were identified (Table S1 and Figure 3). The synthesis and transformation of these compounds under different fermentation conditions not only directly influenced the aroma and taste of the wine but also played a crucial role in its overall flavor balance. The results indicated that compared to single-strain fermentation (JS group), mixed fermentation (JH group) significantly altered the distribution of volatile compounds. This finding suggests that *Pichia kudriavzevii* GAAS-JG-1 has a notable impact on the synthesis and transformation of flavor compounds during sea buckthorn wine fermentation. The following sections provide a detailed classification and analysis of the changes observed in various types of volatile flavor compounds.

#### 3.3.1. Ester Compounds

Esters are primary contributors to the fruity and floral aromas in fruit wines, and their content is significantly influenced by yeast metabolic activity [25]. In this study, a total of 48 ester compounds were identified, with the total ester content in the JH group being significantly higher than that in the JS group, suggesting that *Pichia kudriavzevii* GAAS-JG-1 may enhance esterification metabolism. The content of short-chain esters (e.g., ethyl acetate and ethyl propionate) increased significantly in the JH group. Specifically, ethyl acetate increased from 303.71 μg/L to 4453.12 μg/L, while ethyl propionate rose from 5.18 μg/L to 87.75 μg/L. As key aromatic components in fruit wine, short-chain esters impart distinctive fruity or tropical fruit aromas and play a crucial role in enhancing wine fragrance and sensory appeal [3,26,27]. These results align with previous studies, further supporting the positive effect of mixed fermentation in promoting short-chain ester accumulation and enhancing fruity characteristics. In addition, methyl acetate (11.42 μg/L) and methylthioacetate (23.58 μg/L), which were absent in the JS group, were detected in the JH group. This indicates that the metabolic diversity in the mixed fermentation system may result in a broader spectrum of metabolites [28]. Notably, the content of some long-chain esters (e.g., ethyl hexanoate and ethyl decanoate) decreased slightly in the JH group, dropping from 329.68 and 1148.18 μg/L to 282.36 and 1006.61 μg/L, respectively. Since long-chain esters are generally associated with a wine’s richness and mature fruit aroma, this reduction may contribute to a fresher and more refreshing wine profile. This phenomenon could be attributed to interactions between yeast strains and changes in fermentation conditions during the mixed fermentation process. Several studies have shown that different fermentation strategies can significantly influence the accumulation patterns of volatile aroma compounds in fruit wine, thereby modulating the sensory characteristics of the final product [29,30].

#### 3.3.2. Alcohol Compounds

Alcohol compounds play a key role in shaping wine structure and enhancing flavor complexity. Among them, higher alcohols are important secondary metabolites formed during fermentation; their moderate presence can enhance aroma complexity and improve wine body richness [31]. In this study, 18 higher alcohols were identified, with the overall content of higher alcohols in the JH group exceeding that in the JS group, indicating a superior flavor profile. Specifically, the content of 3-methyl-1-butanol increased from 4841.99 μg/L in the JS group to 5199.39 μg/L in the JH group. This compound has been proven to accumulate during fermentation and impart banana-like fruity notes, potentially enhancing overall flavor complexity and improving the wine’s sensory profile [32]; the phenylethanol levels remained consistently high in both groups (6896.69 μg/L in the JS group and 6636.33 μg/L in the JH group), contributing to rich rose and honey-like aromas that enhance the wine’s floral and sweet characteristics [33]. Notably, 2,3-butanediol (32.17 μg/L) was exclusively detected in the JH group, accumulating significantly during fermentation. This may be attributed to the metabolic characteristics of *Pichia kudriavzevii* GAAS-JG-1 under mixed fermentation conditions. Studies have reported that the production of 2,3-butanediol is typically associated with high yeast fermentation activity and imparts fruity and buttery notes to fruit wine [34]. The presence of this compound in the JH group may therefore contribute to enhancing the wine’s flavor complexity. Previous research suggests that higher alcohols at concentrations below 300 mg/L can optimize wine quality, whereas excessive accumulation (>400 mg/L) may exert a negative impact on flavor [35]. In this study, the higher alcohol content in the JH group remained well below this threshold, indicating a favorable concentration range. This not only prevented adverse effects caused by excessive accumulation but also allowed higher alcohols to play a positive role in enhancing flavor complexity and improving mouthfeel structure. In conclusion, the JH group effectively enhanced the higher alcohol content within an optimal range, improving the aroma complexity and wine body richness while maintaining the overall flavor balance. These results highlight the potential of mixed fermentation to regulate higher alcohol metabolism and optimize the sensory quality of fruit wine.

#### 3.3.3. Acid Compounds

In this study, acid compounds played a crucial role in shaping the flavor profile of sea buckthorn wine through dynamic esterification and hydrolysis equilibrium. Studies have shown that short-chain fatty acids (e.g., hexanoic acid and heptanoic acid) serve as key precursors for ester formation, reacting with alcohols to generate aromatic esters that contribute fruity and floral notes to fruit wine. However, certain volatile acids (e.g., octanoic acid and decanoic acid) can lead to unpleasant, rancid, or irritating sensory attributes when their concentrations exceed specific thresholds, thereby disturbing the overall flavor balance of the wine [36]. A total of seven acid compounds were identified in this study. The analysis revealed that the total acid content in the JH group (mixed fermentation) was significantly lower than that in the JS group (single-fermentation control). Specifically, the concentration of hexanoic acid decreased from 351.11 μg/L in the JS group to 334.03 μg/L in the JH group, indicating that the mixed fermentation strategy effectively regulated acid accumulation, reducing excessive volatile acid content and improving the wine’s mouthfeel complexity and sensory harmony. Notably, 3-methyl-1-butanoic acid exhibited a significant increase in the JH group, rising from 115.74 μg/L to 226.18 μg/L. Previous studies have indicated that certain non-*Saccharomyces* yeasts can metabolize branched-chain amino acids (e.g., leucine) via the Ehrlich pathway, producing 3-methyl-1-butanoic acid as a metabolic byproduct [37]. Since *Pichia kudriavzevii* is an active non-*Saccharomyces* yeast, it may possess a similar metabolic mechanism, promoting the accumulation of 3-methyl-1-butanoic acid during fruit wine fermentation. Although fatty acids are often associated with undesirable odors, research suggests that they are perceptible only when their concentrations exceed 20 mg/L [38]. In this study, the concentrations of all detected acid compounds were significantly below their sensory thresholds, ensuring they would not adversely affect the wine’s flavor. On the contrary, these acids, present in appropriate concentrations, may serve as important flavor precursors that react with alcohols to form aromatic esters, thereby enriching the overall aroma profile of the fruit wine.

#### 3.3.4. Aldehyde and Ketone Compounds

Aldehyde and ketone compounds can impart fruity, floral, and creamy notes to wine when present at appropriate levels; however, excessive concentrations may weaken the perceived intensity of the overall aroma [39]. In this study, three aldehyde and ketone compounds were identified, and different fermentation strategies significantly influenced their accumulation patterns. Among these, 6-methyl-5-hepten-2-one was successfully detected in the fermented sea buckthorn wine, with relatively high concentrations observed in both groups, though no significant difference was found between them. Studies have shown that 6-methyl-5-hepten-2-one is an aromatic compound with fruity and floral characteristics, playing an important role in the overall flavor composition of fruit wine [40]. The nonanal content increased significantly in the JH group (32.53 μg/L) compared to in the JS group (24.72 μg/L). Nonanal is a characteristic compound widely found in spirits and fruit wines, known to enhance floral, fruity, green (grassy), and woody notes [41]. The elevated nonanal content in the JH group may have contributed to increased aroma complexity in the wine. The concentration of damascenone, a compound known to impart fruity and sweet notes to wine, was similar in both groups [42]. These differences suggest that different yeast strains exhibit distinct selectivity in the metabolism of aldehyde and ketone compounds, ultimately influencing the aroma composition and flavor complexity of sea buckthorn wine.

## 4. Conclusions

This study systematically evaluated the tolerance and fermentation characteristics of *Pichia kudriavzevii* GAAS-JG-1, isolated from a naturally fermented apricot system, under various extreme environmental conditions. The results demonstrated that this strain maintained strong growth activity even in an extremely acidic environment (pH 2) and exhibited remarkable tolerance to high ethanol concentrations (16%), high sugar levels (350 g/L), and elevated sulfur dioxide concentrations (500 mg/L). Additionally, *Pichia kudriavzevii* GAAS-JG-1 displayed exceptional growth performance at 35 °C, outperforming all control strains. Further co-fermentation experiments revealed that *Pichia kudriavzevii* GAAS-JG-1, when combined with *Saccharomyces cerevisiae* in sea buckthorn wine fermentation, significantly enhanced the variety and concentration of short-chain esters while moderately regulating the balance of higher alcohols and organic acids. This effectively enhanced the wine’s aroma profile by enriching short-chain esters and balancing higher alcohols and organic acids. Based on these findings, *Pichia kudriavzevii* GAAS-JG-1 demonstrates considerable potential as a fermentation starter for specialty fruit wines and other fermented food products. Future studies should further explore the metabolic pathways and flavor modulation mechanisms of this strain and validate its adaptability and fermentation performance across various fruit wine systems to support its broader application in the food fermentation industry.

## Figures and Tables

**Table 1:** Sequence information of *Pichia kudriavzevii* GAAS-JG-1.

S955-2	GAAS-JG-1	514 bp
GTAGCGGCGAGTGAAGCGGCAAGAGCTCAGATTTGAAATCGTGCTTTGCGGCACGAGTTGTAGATTGCAGGTTGGAGTCTGTGTGGAAGGCGGTGTCCAAGTCCCTTGGAACAGGGCGCCCAGGAGGGTGAGAGCCCCGTGGGATGCCGGCGGAAGCAGTGAGGCCCTTCTGACGAGTCGAGTTGTTTGGGAATGCAGCTCCAAGCGGGTGGTAAATTCCATCTAAGGCTAAATACTGGCGAGAGACCGATAGCGAACAAGTACTGTGAAGGAAAGATGAAAAGCACTTTGAAAAGAGAGTGAAACAGCACGTGAAATTGTTGAAAGGGAAGGGTATTGCGCCCGACATGGGGATTGCGCACCGCTGCCTCTCGTGGGCGGCGCTCTGGGCTTTCCCTGGGCCAGCATCGGTTCTTGCTGCAGGAGAAGGGGTTCTGGAACGTGGCTCTTCGGAGTGTTATAGCCAGGGCCAGATGCTGCGTGCGGGGACCGAGGACTGCGGCCGTGTAGGTCA		

## Data Availability

The original contributions presented in this study are included in the article.

## Supplementary Materials

The following supporting information can be downloaded at https://www.mdpi.com/article/10.3390/microorganisms13071491/s1. Table S1: Changes in volatile flavor compound contents in sea buckthorn wine under different fermentation conditions.
