# Supplementary material for: Stress Tolerance and Contribution to Aroma Profile of Pichia kudriavzevii GAAS-JG-1 Isolated from Apricot Fermentation in Co-Fermentation of Sea Buckthorn Wine
Source: Microorganisms. 2025 Jun 26;13(7):1491. doi: 10.3390/microorganisms13071491 (PMC12299877; doi:10.3390/microorganisms13071491)
Supplement: Supplementary file 1 [file microorganisms-13-01491-s001.zip › microorganisms-3672595-supplementary.pdf]

Supplementary Materials

Table S1. Changes in volatile flavor compound contents in sea buckthorn wine under different fermentation conditions.

No.	CAS	Compound	Concentration(μg/L)	
			JS	JH
A1	79-20-9	Methyl acetate	nd	11.42±0.29
A2	141-78-6	Ethyl acetate	303.71±11.78b	4453.12±162.27a
A3	105-37-3	Ethyl propionate	5.18±0.1b	87.75±2.31a
A4	97-62-1	Ethyl isobutyrate	2.44±0.08b	3.96±0.07a
A5	110-19-0	Isobutyl acetate	nd	12.31±0.19
A6	105-54-4	Ethyl butyrate	12.55±0.24a	13.49±0.21a
A7	7452-79-1	Ethyl 2-methylbutyrate	23.48±0.65a	15.44±0.31b
A8	108-64-5	Ethyl isovalerate	54.99±2.7a	47.49±0.81a
A9	32665-23-9	Isopropyl 3-methylbutanoate	24.52±0.84a	21.28±0.34b
A10	123-92-2	Isoamyl acetate	90.12±2.82b	123.48±2.41a
A11	819-97-6	Sec-butyl butyrate	37.66±1.16a	34.61±0.9a
A12	869-08-9	3-Methylbutanoic acid Sec-butyl ester	110.72±4.51a	101.63±2.5a
A13	2445-67-2	Isobutyl 2-methylbutyrate	26.08±0.92a	24.73±0.48a
A14	589-59-3	Isobutyl isovalerate	152.41±3.18a	157.02±2.53a
A15	106-27-4	Isoamyl butyrate	66.91±1.54a	51.8±1.23b
A16	2445-69-4	2-Methylbutyric acid isobutyl ester	108.99±2.33a	106.48±1.79a
A17	123-66-0	Ethyl hexanoate	329.68±6.59a	282.36±5.25b
A18	51115-64-1	2-Methylbutylbutyrate	57.24±1.58	nd
A19	27625-35-0	Isoamyl 2-methylbutyrate	156.39±3.54a	150.42±3.22a
A20	2445-78-5	2-Methylbutyl 2-methylbutyrate	378.82±10.23a	354.55±6.04a
A21	2445-77-4	2-Methylbutyl isovalerate	4434.21±187.86a	4270.82±101.72a
A22	106-30-9	Ethyl heptanoate	167.74±4.20b	191.48±5.16a
A23	105-79-3	Isobutyl hexanoate	566.74±12.17a	17.24±0.40b
A24	112-06-1	1-heptyl acetate	2.6±0.08b	4.32±0.09a
A25	18267-36-2	Ethyl3-hydroxy-3-methylbutyrate	52.37±1.76a	54.27±1.64a
A26	7779-80-8	Heptanoicacid, isobutyl ester	22.1±0.50a	19.77±0.41b
A27	2441-06-7	2-Hydroxy-3-methylbutanoic acid ethyl ester	18.39±0.74a	17.47±0.48a
A28	106-32-1	Ethyl octanoate	2473.71±85.22a	2322.7±62.26a
A29	10032-13-0	3-Methylbutyric acid hexyl ester	24.32±0.88a	21.1±0.62a
A30	4455-13-4	Ethyl(methylthio)acetate	nd	23.58±0.58
A31	2601-13-0	2-Methylbutyl caproate	229.74±7.20a	167.29±5.20b
A32	5405-41-4	Ethyl 3-hydroxybutyrate	9.48±0.21a	7.26±0.19b
A33	5461-06-3	Isobutyl octanoate	129.87±5.50a	125.82±2.96a
A34	123-29-5	Ethyl nonanoate	573.36±12.93a	529.12±10.61a
A35	5451-80-9	Heptyl pentanoate	16.02±0.77	nd
A36	155195-82-5	Isobutyl 3-octenoate	18.58±0.90a	16.24±0.50a
A37	143-13-5	Nonyl acetate	7.77±0.22b	9.87±0.29a
A38	110-38-3	Ethyl decanoate	1148.18±50.51a	1006.61±33.33a
A39	939-48-0	Isopropyl benzoate	26.02±0.84a	25.31±0.67a
A40	2305-25-1	Ethyl 3-hydroxyhexanoate	10.11±0.38	nd
A41	3306-36-3	Sec-butyl benzoate	363.74±9.71a	356.39±10.10a
A42	101-97-3	Ethyl phenylacetate	66.98±2.34a	66.4±1.29a
A43	120-50-3	2-Methylpropyl benzoate	48.49±1.00a	50.38±1.19a
A44	103-45-7	Phenethyl acetate	110.08±4.54b	128.11±3.80a

A45	103-38-8	Benzyl 3-methyl butyrate	15.57±0.78	nd
A46	94-46-2	Isoamyl benzoate	276.31±12.33a	277.05±8.75a
B47	67-63-0	Isopropyl alcohol	21.41±0.81b	26.49±0.85a
B48	78-92-2	2-Butanol	234.19±8.97a	235.74±7.45a
B49	71-23-8	1-Propanol	22.91±1.23b	28.61±0.95a
B50	78-83-1	2-Methyl-1-propanol	773.93±24.74a	760.75±17.51a
B51	71-36-3	1-Butanol	8.91±0.25a	9.09±0.26a
B52	123-51-3	3-Methyl-1-butanol	4841.99±171.42a	5199.39±205.31a
B53	75-65-0	2-Methyl-2-Propanol	7.76±0.21a	6.87±0.17b
B54	111-27-3	1-Hexanol	43.33±2.24a	33.09±0.59b
B55	5271-38-5	2-(Methylthio)ethanol	237.73±13.06a	206.72±10.16a
B56	78-70-6	Linalool	243.39±11.07a	244.17±6.63a
B57	111-87-5	1-Octanol	220.38±6.84a	212.25±5.19a
B58	19132-06-0	2,3-Butanediol	nd	32.17±0.69
B59	562-74-3	Terpinen-4-ol	39.45±1.87a	31.31±0.85b
B60	29957-43-5	3,7-Dimethyl-1,5,7-octatrien-3-ol	57.51±2.90a	60.65±2.31a
B61	143-08-8	1-Nonanol	452.41±15.65a	471.7±14.32a
B62	98-55-5	à-Terpineol	91.31±3.73a	86.37±2.44a
B63	505-10-2	3-(Methylthio)-1-propanol	10.44±0.44b	20.19±0.59a
B64	60-12-8	Phenylethyl alcohol	6896.69±218.08a	6636.33±217.73a
C65	625-08-1	3-Hydroxy-3-methyl-butanoic acid	70.5±2.61a	68.61±2.32a
C66	503-74-2	3-Methylbutanoic acid	115.74±5.67b	226.18±7.43a
C67	142-62-1	Hexanoic acid	351.11±14.52a	334.03±9.84a
C68	111-14-8	Heptanoic acid	nd	50.73±1.35
C69	124-07-2	Octanoic acid	145.82±6.56a	136.2±3.71a
D70	110-93-0	6-methyl-5-Hepten-2-one	252.02±10.38a	230.91±8.12a
D71	124-19-6	Nonanal	24.72±1.08b	32.53±0.94a
D72	23726-93-4	Beta-damascenone	11.1±0.36a	10.24±0.29a

Note: Different superscript letters indicate significant differences between groups ($p< 0.05$), while the same superscript letters indicate non-significant differences between groups ($p> 0.05$). -: not detected. JS: Monoculture fermentation of *Saccharomyces cerevisiae* (CICC 32168); JH: *Saccharomyces cerevisiae* (CICC 32168) was inoculated initially, and *Pichia kudriavzevii* GAAS-JG-1 was inoculated after 24 h.
